# Amniotic membrane transplantation for wound dehiscence after deep lamellar keratoplasty: a case report

**DOI:** 10.1186/1752-1947-1-28

**Published:** 2007-06-13

**Authors:** Tetsuya Kawakita, Tamaki Sumi, Murat Dogru, Kazuo Tsubota, Jun Shimazaki

**Affiliations:** 1 Department of Ophthalmology, Tokyo Dental College Ichikawa General Hospital, Chiba, Japan, 272-8513; 2 Department of Ophthalmology, Keio University, Tokyo, Japan, 160-8582

## Abstract

**Purpose:**

To report amniotic membrane (AM) transplantation in a patient with wound dehiscence 5 months after deep lamellar keratoplasty (DLKP)

**Methods:**

The patient was an 84-year-old Japanese man who had undergone right DLKP 5 months earlier for central corneal scarring due to recurrent stromal herpetic keratitis. He developed wound dehiscence with corneal stromal melting due to recurrence of stromal herpes in both the donor and recipient sites. "AM roll-in filling technique" and AM patching were performed.

**Results:**

Following AM transplantation, stromal inflammation subsided and complete epithelization occurred within 10 days of surgery.

At 8 months postoperatively, biomicroscopy revealed stable wound apposition or stromal gain. Following AM transplantation, stromal inflammation subsided and complete epithelialization was achieved within 10 days after surgery.

**Conclusion:**

AM transplantation may offer an effective treatment modality for herpetic corneal wound dehiscence after DLKP.

## Background

AM transplantation has been reported to be an effective ocular surface reconstruction procedure in the treatment of corneal erosions, central or peripheral ulcers and perforations, as such membranes can decrease inflammation, promote corneal epithelialization and provide corneal stromal substrate.[1,2] We report AM transplantation in a patient with wound dehiscence 5 months after deep lamellar keratoplasty (DLKP).

## Case presentation

**Figure 1:**
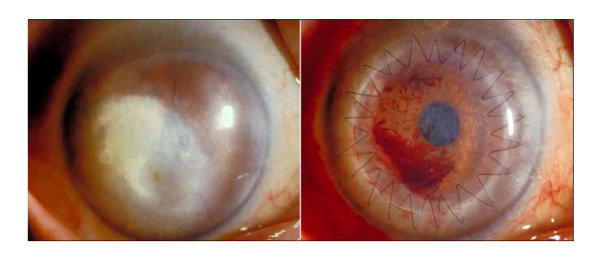
**Left, preoperative appearance showing lipid deposition covering pupil**. Right, postoperative appearance 2 weeks after DLKP. There is blood in the interface between graft and host.

An 84-year-old Japanese man was referred to our hospital for keratoplasty-due to central corneal opacity and peripheral corneal neovascularization with lipid deposition in the right eye (Figure 1A). His medical history showed that laboratory culture and serological tests had revealed recurrent herpetic keratitis in that eye. At his initial visit, the best corrected visual acuities (BCVA) were 12/200 OD and 20/20 OS.

DLKP with single running 10-0 nylon sutures was performed (Figure 1, right). Complete graph epithelization was achieved within 5 days. In addition to 0.1% topical dexamethasone qid (Sanbethasone^®^, Santen) and levofuroxacine eyedrops qid (Cravit^®^, Santen) for 5 months, the patient was prescribed 1000 mg/day oral acyclovir (Zovirax^®^, Glaxo Smith Kline), to be commenced the day prior to the operation and continued for 10 days to prevent herpetic recurrence.

**Figure 2:**
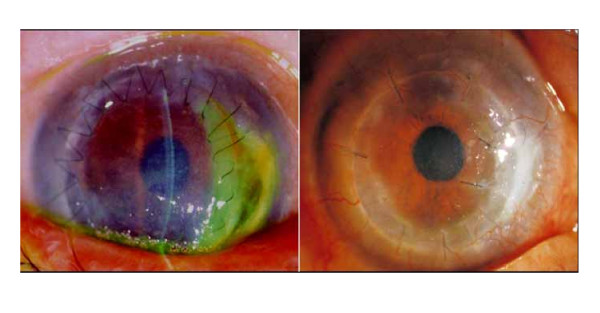
**Left, postoperative appearance 5 months after DLKP showing ulceration, stromal melting, wound dehiscence and iris incarceration**. Right, postoperative appearance 2 weeks after AMT.

The corneal graft remained in good condition with recovery of BCVA to 20/100 until the fifth postoperative month, at which time the patient was readmitted with decreased vision and right ocular pain. Examination revealed stromal herpetic keratitis, stromal melting and wound dehiscence with descemetocele at between 2 and 4 o'clock to the donor-recipient apposition site. (Figure 2, left) The anterior chamber was shallow, and incarceration of the iris was observed. The patient was prescribed 1000 mg peroral acyclovir and ointment five times a day. Due to the development of corneal perforation and unavailability of donor corneal tissue, running sutures were replaced with interrupted sutures, and frozen AM trimmed to fit the site was transplanted with a "roll-in filling technique", i.e., roll-in AM was used to provide wound apposition without sutures, while a second AM patch was used to cover the melting area with interrupted sutures. (Figure2, right, AMT indicated by arrow). Preservative-free hyaluronate and topical antibiotic eye drops were prescribed qid. Acyclovir ointment was prescribed five times a day for 3 months. Following AM transplantation, stromal inflammation subsided and complete epithelization was achieved within 10 days of surgery. At 8 months postoperatively, biomicroscopy revealed stable wound apposition and stromal gain.

## Discussion

Postkeratoplasty oral acyclovir prophylaxis has been reported to prevent recurrences. In our opinion, the wound perforation seen here was a result of insufficient prophylaxis with recurrence. AM transplantation has been widely reported to be an efficient procedure for central and peripheral corneal erosion, ulceration and perforations. The beneficial effectsof this approach result from the presence of a rich extracellular matrix and collagen which provide a stromal substrate as in our case and anti-inflammatory properties arising from entrapment of inflammatory cells, the presence of various growth factors, inhibition of proteinase activity, and decrease of lipid peroxidation.[3] AM patch has also been reported to be effective in acute ulcerative and necrotizing herpetic stromal keratitis[4] due toreduction of gelatinolytic activity of MMP-9 and increased expression of TIMP-1.[5] These properties may have been responsible for the effective suppression of herpetic inflammation seen in this particular case.

AM has been commomly used to repair areas of corneal stromal loss by mutilayered AM, but which technique is difficult to apply for wound dehiscence because of shape of stromal loss. Our modified "AM roll-in filling technique" can provide compact and dense spacer for such stromal loss site. We have reported the successful application of AM in wound dehiscence and herpetic stromal melting after DLKP. We have also demonstrated the usefulness of the "AM roll-in filling technique" for such patients. Due to availability of corneal donor, this technique could be used as a first choice in such situation.

## Abbreviations

AM; amniotic membrane, AMT; amniotic membrane transplantation, BCVA; the best corrected visual acuities, DLKP; deep lamellar keratoplasty, MMP; matrix metalloproteinase, TIMP; tissue inhibitor of metalloproteinase

## Competing interests

The author(s) declare that they have no competing interests.

## Authors' contributions

TK: Analysis and interpretation, writing the draft manuscript

TS: Data collection, provision of patient materials

MD: Provision of patient material, critical revision of the article

KT: Provision of materials and resources

JS: Conception and design, analysis and interpretation

All of the authors read and approved the final manuscript.
